# To Have or Not To Have a Pet for Better Health?

**DOI:** 10.1371/journal.pone.0000109

**Published:** 2006-12-27

**Authors:** Leena K. Koivusilta, Ansa Ojanlatva

**Affiliations:** 1 Institutions and Social Mechanisms (IASM) Consortium, University of Turku, Turku, Finland; 2 Department of Teacher Education and Institute of Biomedicine, Center for Reproductive and Developmental Medicine, University of Turku, Turku, Finland; 3 Turku City Hospital, Turku, Finland; James Cook University, Australia

## Abstract

**Background:**

Pet ownership is thought to have health benefits, but not all scientific explorations have been founded on proper applications of representative samples or statistically correct methodologies. Databanks have been too small for proper statistical analyses; or, instead of a random sample, participation has been voluntary. The direction of causality has been evaluated incorrectly or control of relevant factors noted deficient. This study examined the associations of pet ownership with perceived health and disease indicators by taking into account socio-demographic background factors together with health risk factors, including exercise.

**Methodology/Principal Findings:**

The present study used baseline data from the 15-year Health and Social Support Study (the HeSSup Study). The Finnish Population Register Centre was used to draw population-based random samples stratified according to gender and four age groups (20–24, 30–34, 40–44, and 50–54 years). A total of 21,101 working-aged Finns responded to the baseline survey questionnaire of the 15-year HeSSup Study in 1998. Ordinal and binary logistic regression was used to analyze the cross-sectional data. Pet ownership was associated with poor rather than good perceived health. BMI surfaced as the risk factor most strongly associated with pet ownership.

**Conclusions/Significance:**

Pet owners set in their ways and getting older were found to have a slightly higher BMI than the rest. Additional research is needed for the testing of hypotheses involving effects of pet ownership with various health dimensions within population groups that are composed of different kinds of background characteristics.

## Introduction

Pet ownership is thought to have health benefits, but not all scientific explorations have been founded on proper applications of representative samples or statistically correct methodologies. Databanks have been too small for proper statistical analyses or instead of a random sample, participation has been voluntary. The direction of causality has been evaluated incorrectly or control of relevant factors noted deficient. Weaknesses have been stated e.g., with less cardiovascular mortality among young people or variance in measuring pet ownership [1].

Several countries have pioneered pet-related research at the national level [2]–[5]. The first study illustrated moderate associations of pet ownership with lower blood pressure and less risk of heart attack or stroke with volunteer participants [6]–[7]. An intervention study measured the impact of adverse life events and established that those having a pet coped significantly better than those not having one [5]. Elderly pet owners without immediate medical attention coped with stressful life events better when they had a pet [8]. No direct statistically significant association was observed between pet ownership and changes in psychological well-being [9].

A telephone interview associated pet ownership with better physical and mental health, fewer visits to a physician, and fewer medications involving problems of blood pressure, sleep, cholesterol, or a heart problem [10]–[11]. Pet ownership was connected with better self-reported physical and psychological health and with fewer visits to see a physician when the most important demographic variables were controlled [12]. Findings of large and representative follow-up studies controlling for important disease risk factors have demonstrated reduced uses of physician services [4].

At least some level of evidence exists that having a pet contributes to reduced cardiovascular diseases or evident risk factors [13]–[14]. A10-month intervention study (with follow-up) that also involved some volunteers living with a pet indicated that physical and mental health of the pet owners improved and that they moved about more [2]. Elongated survival periods of pet owners have been observed after a heart attack [15]–[17].

Cross-sectional surveys have not indicated associations of pet ownership with cardiovascular health benefits per se. Pet owners were recorded to have higher diastolic blood pressures [18] or otherwise poorer health [19] than those without pets. Studies on potential anxiety-reducing effects of pets [20]–[21] have yielded contradictory results [22]–[23].

Up to this point, research has often used non-representative samples of specific population groups such as aging individuals or people with particular diseases. Samples have also been small making multivariate analyses impossible. Health has also mostly been examined with cardiovascular diseases, and consequently, a variety of health indicators was seen necessary to be included. The present study aims to address shortcomings of previous research while it concentrates on a representative sample of working aged individuals.

The aim of the present study was to examine associations of pet ownership with perceived health and disease indicators within general working-aged population groups in order to provide comparison data for past selective groups involving old age and/or poor health. Socio-demographic factors together with health risk factors, including exercise were taken into account.

## Methods

The Finnish Population Register Centre was used to draw population-based random samples stratified according to gender and 4 age groups (20–24, 30–34, 40–44, and 50–54 years). The age groups were selected in order to have a wide age distribution and a concentration of specific age groups, and to identify generational divergences. A special mailing service distributed the survey questionnaire together with consent forms to 52,739 eligible participants in 1998, collected them with signatures and used automatic recording of the dataset. The response rate was 40.8% following one reminder. A total of 21,101 individuals (40%) remained available for the analyses. Representativeness of the study has been reported elsewhere [24].

According to the Finnish law, an approval from the university ethics committee was not necessary because of healthy subjects. Informed consent was adequate with signature for linkage of their personal information via registries.

### Outcome Variables

Outcome Variables

1. Perceived health status (good, fair, poor).
2. Disease indicators were mapped by asking whether or not a physician had ever said that the participants had any of the disease indicators also listed in the Finnish ICD10 classification. Disease indicators were chronic bronchitis or emphysema of the lung, asthma, allergic rhinitis (e.g., hay fever), high blood pressure, hypertension, high cholesterol, diabetes, myocardial infarction, angina pectoris, arterial fibrillation or flutter, stroke, other disorder of the brain circulation, gastric/duodenal ulcer, liver disease, kidney disease, rheumatoid arthritis, osteoarthritis, sciatica, grey cataract or glaucoma, migraine, epilepsy, brain damage (more serious than concussion), meningitis or cerebrospinal meningitis, other brain or neurological disease, depression, panic attack, anorexia or bulimia, other mental disturbance, malignant growth (cancer). A sum of disease indicators contained all those a person had with the exception of asthma and allergic rhinitis that had a reverse order in their associations with pet ownership. Values were grouped into 4 categories based on the quartile distribution.

### Primary Explanatory Variable

The question inquired about ‘now’ having/not having a pet (cat, dog, other animal) with 3 response alternatives (yes, no/not wanted, no/impossible to keep). Two response alternatives (yes or no/not wanted/impossible to keep) were used when analyzing associations with the socio-demographic variables. A new variable was created (dog plus additional pets within the same family vs. other pets) in effort to establish whether dog owners were different from the others and/or potentially moved about in health promotion terms more than those not having a dog.

### Health Risk Factors and Physical Activity

1. Present smoker: does not presently smoke, smokes occasionally, smokes regularly.
2. Intake of alcohol: 0 g/week, 0–22 g/week (woman) or 0–33 g/week (man), 23–189 g/week (woman) or 34–279 g/week (man), over 189g/week (woman) or over 279 g/week (man).
3. Strenuousness of physical exercise (an activity metabolic equivalent, MET, calculated as an index with duration and exertion) [25]: ≥2 MET hours of daily physical activity, <2 MET hours of daily physical activity.
4. Exercise-related hobbies: At least 1–3 times per month, less often.
5. Body mass index (the cut-off point ≥27 ∼10% above the upper limit for normal weight or halfway between overweight and obesity [26], the limit will separate and retain the reasonably overweight persons above it): ≥27, <27.

### Socio-demographic Background

The personal background characteristics are included in Table 1. The classification of basic and professional education was according to the Finnish educational system [27]. Vocational education included no vocational education, a vocational education course (minimum of 4 months) or vocational school/apprenticeship training, graduation from vocational institute/college, graduation from university/higher education. A marriage-like relationship refers to a circumstance when two heterosexual people live together out-of-wedlock.

**Table 1:**
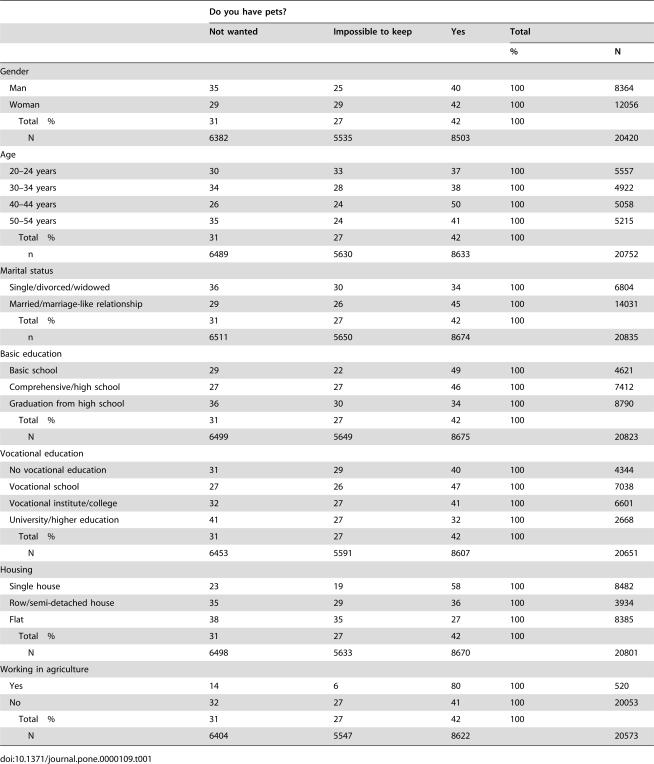
Distributions (%) of those having or not having a pet by their socio-demographic background.

	Do you have pets?				
	Not wanted	Impossible to keep	Yes	Total	
				%	N
Gender					
Man	35	25	40	100	8364
Woman	29	29	42	100	12056
Total %	31	27	42	100	
N	6382	5535	8503		20420
Age					
20–24 years	30	33	37	100	5557
30–34 years	34	28	38	100	4922
40–44 years	26	24	50	100	5058
50–54 years	35	24	41	100	5215
Total %	31	27	42	100	
n	6489	5630	8633		20752
Marital status					
Single/divorced/widowed	36	30	34	100	6804
Married/marriage-like relationship	29	26	45	100	14031
Total %	31	27	42	100	
n	6511	5650	8674		20835
Basic education					
Basic school	29	22	49	100	4621
Comprehensive/high school	27	27	46	100	7412
Graduation from high school	36	30	34	100	8790
Total %	31	27	42	100	
N	6499	5649	8675		20823
Vocational education					
No vocational education	31	29	40	100	4344
Vocational school	27	26	47	100	7038
Vocational institute/college	32	27	41	100	6601
University/higher education	41	27	32	100	2668
Total %	31	27	42	100	
N	6453	5591	8607		20651
Housing					
Single house	23	19	58	100	8482
Row/semi-detached house	35	29	36	100	3934
Flat	38	35	27	100	8385
Total %	31	27	42	100	
N	6498	5633	8670		20801
Working in agriculture					
Yes	14	6	80	100	520
No	32	27	41	100	20053
Total %	31	27	42	100	
N	6404	5547	8622		20573

### Statistical Methods

Associations were examined with Pearson χ2 test (Tables 1 to [Table pone-0000109-t002] [Table pone-0000109-t003] 4), univariate and multivariate ordinal or binary logistic regression analyses, depending on the scale of measurement of the outcome variable [28] (Table 5). Multivariate analyses utilized those socio-demographic and risk factor indicators that formed univariate associations with a given outcome variable. Ordinal logistic regression analysis is an extension of a binary logistic regression, and allows for modeling of polytomous ordinal responses on a set of predictors. The reference class was formed as the one that was theoretically least likely to be associated with poor perceived health or with several different disease indicators. Statistical Package for Social Sciences (SPSS 12.0 for Windows) was used.

**Table 2 pone-0000109-t002:**
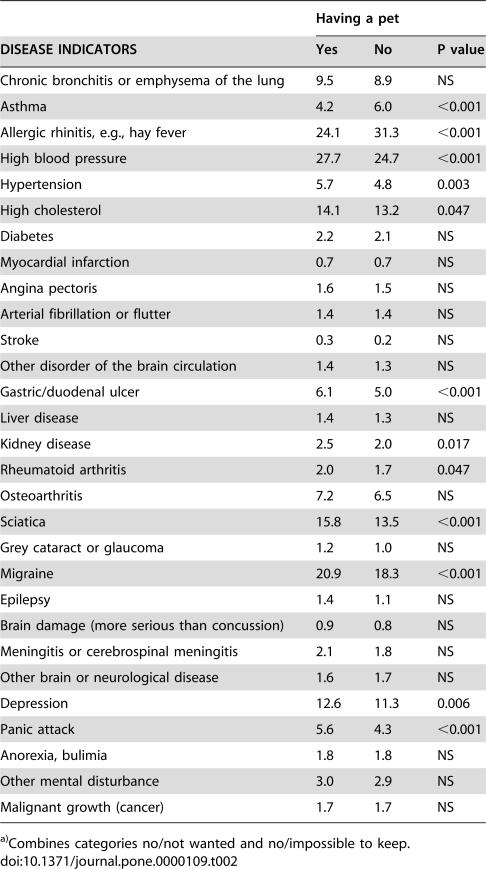
Distribution of disease indicators (%) by groups of having/not having a peta).

	Having a pet		
DISEASE INDICATORS	Yes	No	P value
Chronic bronchitis or emphysema of the lung	9.5	8.9	NS
Asthma	4.2	6.0	<0.001
Allergic rhinitis, e.g., hay fever	24.1	31.3	<0.001
High blood pressure	27.7	24.7	<0.001
Hypertension	5.7	4.8	0.003
High cholesterol	14.1	13.2	0.047
Diabetes	2.2	2.1	NS
Myocardial infarction	0.7	0.7	NS
Angina pectoris	1.6	1.5	NS
Arterial fibrillation or flutter	1.4	1.4	NS
Stroke	0.3	0.2	NS
Other disorder of the brain circulation	1.4	1.3	NS
Gastric/duodenal ulcer	6.1	5.0	<0.001
Liver disease	1.4	1.3	NS
Kidney disease	2.5	2.0	0.017
Rheumatoid arthritis	2.0	1.7	0.047
Osteoarthritis	7.2	6.5	NS
Sciatica	15.8	13.5	<0.001
Grey cataract or glaucoma	1.2	1.0	NS
Migraine	20.9	18.3	<0.001
Epilepsy	1.4	1.1	NS
Brain damage (more serious than concussion)	0.9	0.8	NS
Meningitis or cerebrospinal meningitis	2.1	1.8	NS
Other brain or neurological disease	1.6	1.7	NS
Depression	12.6	11.3	0.006
Panic attack	5.6	4.3	<0.001
Anorexia, bulimia	1.8	1.8	NS
Other mental disturbance	3.0	2.9	NS
Malignant growth (cancer)	1.7	1.7	NS

a) Combines categories no/not wanted and no/impossible to keep.

**Table 3 pone-0000109-t003:**
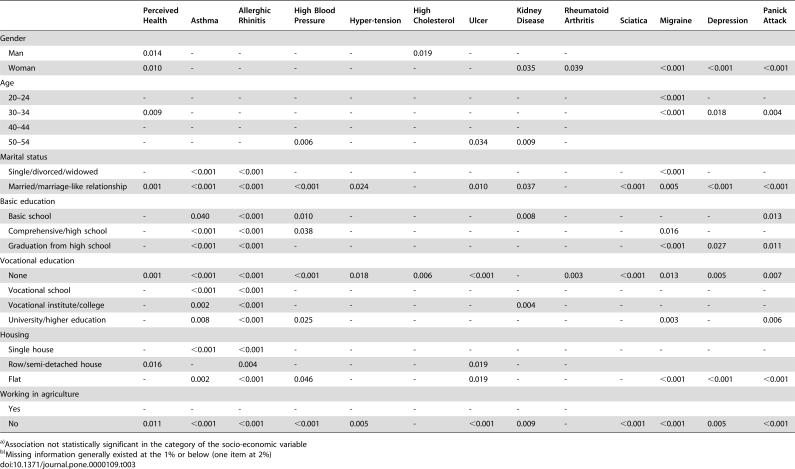
Statistical significancies for associations between pet ownership (p-values in χ^2^ tests) and the health/disease indicators in the categories of the socio-demographic variablesa) b)

	Perceived Health	Asthma	Allerghic Rhinitis	High Blood Pressure	Hyper-tension	High Cholesterol	Ulcer	Kidney Disease	Rheumatoid Arthritis	Sciatica	Migraine	Depression	Panick Attack
Gender													
Man	0.014	-	-	-	-	0.019	-	-	-	-	-	-	-
Woman	0.010	-	-	-	-	-	-	0.035	0.039	-	<0.001	<0.001	<0.001
Age													
20–24	-	-	-	-	-	-	-	-	-	-	<0.001	-	-
30–34	0.009	-	-	-	-	-	-	-	-	-	<0.001	0.018	0.004
40–44	-	-	-	-	-	-	-	-	-	-	-	-	-
50–54	-	-	-	0.006	-	-	0.034	0.009	-	0.001	-	-	-
Marital status													
Single/divorced/widowed	-	<0.001	<0.001	-	-	-	-	-	-	-	<0.001	-	-
Married/marriage-like relationship	0.001	<0.001	<0.001	<0.001	0.024	-	0.010	0.037	-	<0.001	0.005	<0.001	<0.001
Basic education													
Basic school	-	0.040	<0.001	0.010	-	-	-	0.008	-	-	-	-	0.013
Comprehensive/high school	-	<0.001	<0.001	0.038	-	-	-	-	-	-	0.016	-	-
Graduation from high school	-	<0.001	<0.001	-	-	-	-	-	-	-	<0.001	0.027	0.011
Vocational education													
None	0.001	<0.001	<0.001	<0.001	0.018	0.006	<0.001	-	0.003	<0.001	0.013	0.005	0.007
Vocational school	-	<0.001	<0.001	-	-	-	-	-	-	-	-	-	-
Vocational institute/college	-	0.002	<0.001	-	-	-	-	0.004	-	-	-	-	-
University/higher education	-	0.008	<0.001	0.025	-	-	-	-	-	-	0.003	-	0.006
Housing													
Single house	-	<0.001	<0.001	-	-	-	-	-	-	-	-	-	-
Row/semi-detached house	0.016	-	0.004	-	-	-	0.019	-	-	-	0.034	0.012	-
Flat	-	0.002	<0.001	0.046	-	-	0.019	-	-	-	<0.001	<0.001	<0.001
Working in agriculture													
Yes	-	-	-	-	-	-	-	-	-	-	-	-	-
No	0.011	<0.001	<0.001	<0.001	0.005	-	<0.001	0.009	-	<0.001	<0.001	0.005	<0.001

a) Association not statistically significant in the category of the socio-economic variable

b) Missing information generally existed at the 1% or below (one item at 2%)

**Table 4:**
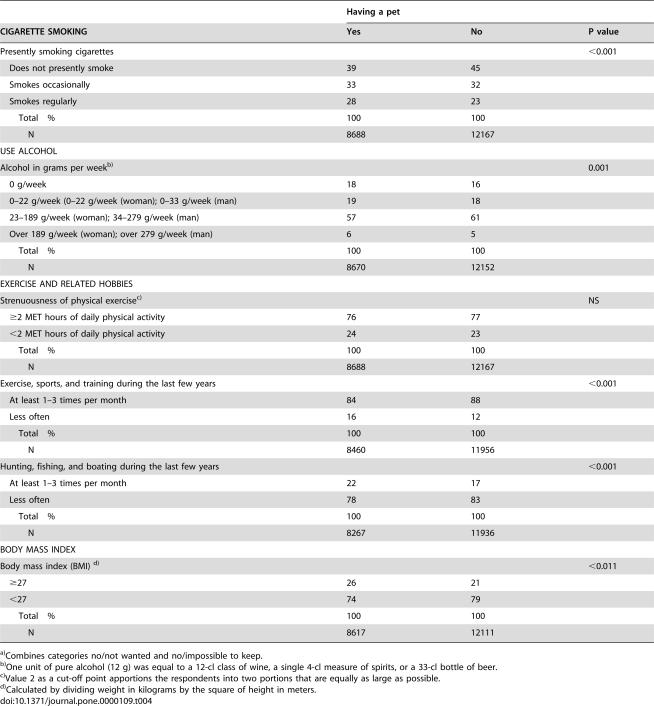
Distribution of risk factors of disease by having/not having a peta).

	Having a pet		
CIGARETTE SMOKING	Yes	No	P value
Presently smoking cigarettes			<0.001
Does not presently smoke	39	45	
Smokes occasionally	33	32	
Smokes regularly	28	23	
Total %	100	100	
N	8688	12167	
USE ALCOHOL			
Alcohol in grams per weekb)			0.001
0 g/week	18	16	
0–22 g/week (0–22 g/week (woman); 0–33 g/week (man)	19	18	
23–189 g/week (woman); 34–279 g/week (man)	57	61	
Over 189 g/week (woman); over 279 g/week (man)	6	5	
Total %	100	100	
N	8670	12152	
EXERCISE AND RELATED HOBBIES			
Strenuousness of physical exercisec)			NS
≥2 MET hours of daily physical activity	76	77	
<2 MET hours of daily physical activity	24	23	
Total %	100	100	
N	8688	12167	
Exercise, sports, and training during the last few years			<0.001
At least 1–3 times per month	84	88	
Less often	16	12	
Total %	100	100	
N	8460	11956	
Hunting, fishing, and boating during the last few years			<0.001
At least 1–3 times per month	22	17	
Less often	78	83	
Total %	100	100	
N	8267	11936	
BODY MASS INDEX			
Body mass index (BMI) d)			<0.011
≥27	26	21	
<27	74	79	
Total %	100	100	
N	8617	12111	

a) Combines categories no/not wanted and no/impossible to keep.

b) One unit of pure alcohol (12 g) was equal to a 12-cl class of wine, a single 4-cl measure of spirits, or a 33-cl bottle of beer.

c) Value 2 as a cut-off point apportions the respondents into two portions that are equally as large as possible.

d) Calculated by dividing weight in kilograms by the square of height in meters.

## Results

Slightly more women than men had pets, whereas more men than women did not want pets at all (Table 1). People living in couple relationships had pets more often. The frequency of having pets increased as the level of basic or vocational education went down. Overall, 40–44-year-olds had the most pets and 20–24-year-olds had the least pets. Pets were most often found in single houses and in the flats least often. Those working in agriculture were pet owners clearly more often than the rest.

### Perceived Health

A total of 80% of those having and 82% of those not having pets reported good health (p = 0.001). The proportions of respondents with poor perceived health were equally large (4%) in both groups. The associations of poor perceived health with pet ownership were present among both genders (Table 3), among 30–34-year-olds, among those in couple relationships, among those with no vocational training, living in a row/semi-detached house, or not working in agriculture.

### Disease indicators

Asthma, allergic rhinitis, high blood pressure, hypertension, high cholesterol, ulcer, kidney disease, rheumatoid arthritis, sciatica, migraine, depression, and panic attack were statistically significantly associated with pet ownership but the differences were small between groups. With the exception of asthma/allergic rhinitis, there were more disease indicators among pet owners than among those not having pets (Table 2).

The associations of having pets and asthma/allergic rhinitis, and migraine were clearly independent of several background factors (Table 3). People with no vocational education and training but some highly educated ones had several associations with disease indicators as well. Associations of pet ownership with physical health were typical for older and with emotional health for younger people. Many of the associations were found among people with relationships, whereas none were observed among people working in agriculture.

### Health Risk Factors and Physical Activity

Pet owners smoked cigarettes more often but consumed alcohol less often than those not having pets (Table 4). A higher BMI was associated with pet ownership. Strenuous exercise was equally represented between the pet groups. Pet owners spent slightly less spare time in sports activities but went hunting, fishing, or boating more often.

The associations of having pets with all risk factors were independent of gender. The associations were about the same in all age groups, but the one with strenuous exercise disappeared when controlled with age, marital status or basic education. Strenuous exercise was typical of 20–24-year-olds, those not in couple relationship, and those having graduated from high school, while they also had pets less often (p<0.001 each). Associations of having pets with health risk factors were present in almost all categories of educational variables and housing. Except for strenuousness of exercise, all associations of risk factors with pet ownership were statistically significant outside agriculture alone.

### Logistic Regression Models

#### Perceived Health

The cumulative odds ratio measuring the association of pet ownership with perceived health deviated only slightly from 1.0 but was statistically significant (Table 5). Pet ownership was associated with poor rather than good perceived health. Becoming older, having a low level of basic or vocational education, being a man, being single, divorced, or widowed, living in other than a single house, and working in agriculture seemed to indicate poor perceived health. All listed health risk factors, particularly the exercise-related ones were associated with perceived health in the univariate analyses.

**Table 5:**
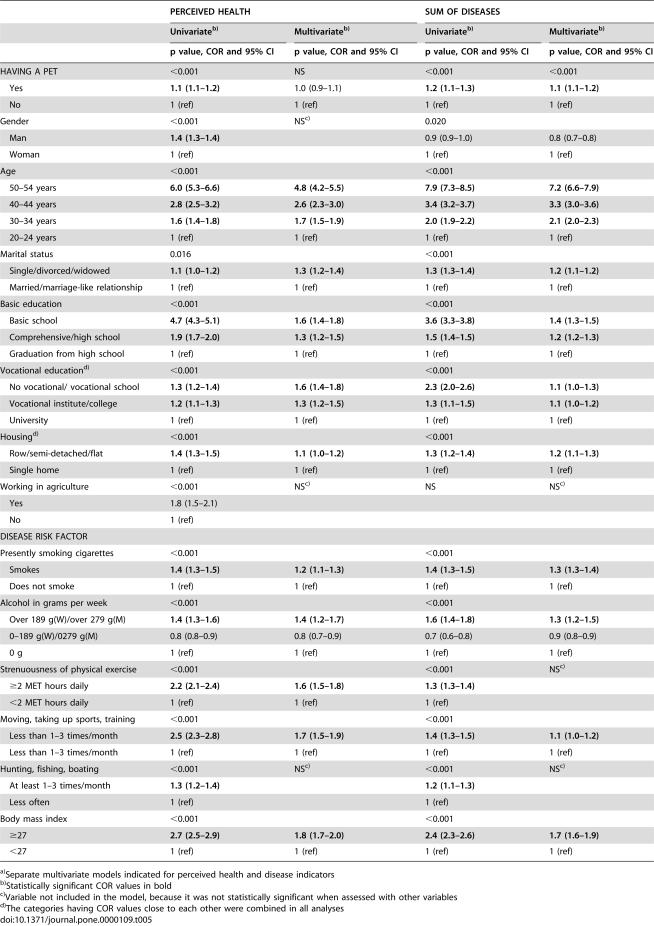
Univariate and multivariate associationsa) of poor perceived health and sum of disease indicators (outcome variables) with pet ownership, socio-demographic background and health risk factors

	PERCEIVED HEALTH		SUM OF DISEASES	
	Univariateb)	Multivariateb)	Univariateb)	Multivariateb)
	p value, COR and 95% CI	p value, COR and 95% CI	p value, COR and 95% CI	p value, COR and 95% CI
HAVING A PET	<0.001	NS	<0.001	<0.001
Yes	**1.1 (1.1–1.2)**	1.0 (0.9–1.1)	**1.2 (1.1–1.3)**	**1.1 (1.1–1.2)**
No	1 (ref)	1 (ref)	1 (ref)	1 (ref)
Gender	<0.001	NSc)	0.020	
Man	**1.4 (1.3–1.4)**		0.9 (0.9–1.0)	0.8 (0.7–0.8)
Woman	1 (ref)		1 (ref)	1 (ref)
Age	<0.001		<0.001	
50–54 years	**6.0 (5.3–6.6)**	**4.8 (4.2–5.5)**	**7.9 (7.3–8.5)**	**7.2 (6.6–7.9)**
40–44 years	**2.8 (2.5–3.2)**	**2.6 (2.3–3.0)**	**3.4 (3.2–3.7)**	**3.3 (3.0–3.6)**
30–34 years	**1.6 (1.4–1.8)**	**1.7 (1.5–1.9)**	**2.0 (1.9–2.2)**	**2.1 (2.0–2.3)**
20–24 years	1 (ref)	1 (ref)	1 (ref)	1 (ref)
Marital status	0.016		<0.001	
Single/divorced/widowed	**1.1 (1.0–1.2)**	**1.3 (1.2–1.4)**	**1.3 (1.3–1.4)**	**1.2 (1.1–1.2)**
Married/marriage-like relationship	1 (ref)	1 (ref)	1 (ref)	1 (ref)
Basic education	<0.001		<0.001	
Basic school	**4.7 (4.3–5.1)**	**1.6 (1.4**–**1.8)**	**3.6 (3.3–3.8)**	**1.4 (1.3–1.5)**
Comprehensive/high school	**1.9 (1.7–2.0)**	**1.3 (1.2–1.5)**	**1.5 (1.4–1.5)**	**1.2 (1.2–1.3)**
Graduation from high school	1 (ref)	1 (ref)	1 (ref)	1 (ref)
Vocational educationd)	<0.001		<0.001	
No vocational/ vocational school	**1.3 (1.2–1.4)**	**1.6 (1.4–1.8)**	**2.3 (2.0–2.6)**	**1.1 (1.0–1.3)**
Vocational institute/college	**1.2 (1.1–1.3)**	**1.3 (1.2–1.5)**	**1.3 (1.1–1.5)**	**1.1 (1.0–1.2)**
University	1 (ref)	1 (ref)	1 (ref)	1 (ref)
Housingd)	<0.001		<0.001	
Row/semi-detached/flat	**1.4 (1.3–1.5)**	**1.1 (1.0–1.2)**	**1.3 (1.2–1.4)**	**1.2 (1.1–1.3)**
Single home	1 (ref)	1 (ref)	1 (ref)	1 (ref)
Working in agriculture	<0.001	NSc)	NS	NSc)
Yes	1.8 (1.5–2.1)			
No	1 (ref)			
DISEASE RISK FACTOR				
Presently smoking cigarettes	<0.001		<0.001	
Smokes	**1.4 (1.3–1.5)**	**1.2 (1.1–1.3)**	**1.4 (1.3–1.5)**	**1.3 (1.3–1.4)**
Does not smoke	1 (ref)	1 (ref)	1 (ref)	1 (ref)
Alcohol in grams per week	<0.001		<0.001	
Over 189 g(W)/over 279 g(M)	**1.4 (1.3–1.6)**	**1.4 (1.2–1.7)**	**1.6 (1.4–1.8)**	**1.3 (1.2–1.5)**
0–189 g(W)/0279 g(M)	0.8 (0.8–0.9)	0.8 (0.7–0.9)	0.7 (0.6–0.8)	0.9 (0.8–0.9)
0 g	1 (ref)	1 (ref)	1 (ref)	1 (ref)
Strenuousness of physical exercise	<0.001		<0.001	NSc)
≥2 MET hours daily	**2.2 (2.1–2.4)**	**1.6 (1.5–1.8)**	**1.3 (1.3–1.4)**	
<2 MET hours daily	1 (ref)	1 (ref)	1 (ref)	
Moving, taking up sports, training	<0.001		<0.001	
Less than 1–3 times/month	**2.5 (2.3–2.8)**	**1.7 (1.5–1.9)**	**1.4 (1.3–1.5)**	**1.1 (1.0–1.2)**
Less than 1–3 times/month	1 (ref)	1 (ref)	1 (ref)	1 (ref)
Hunting, fishing, boating	<0.001	NSc)	<0.001	NSc)
At least 1–3 times/month	**1.3 (1.2–1.4)**		**1.2 (1.1–1.3)**	
Less often	1 (ref)		1 (ref)	
Body mass index	<0.001		<0.001	
≥27	**2.7 (2.5–2.9)**	**1.8 (1.7–2.0)**	**2.4 (2.3–2.6)**	**1.7 (1.6–1.9)**
<27	1 (ref)	1 (ref)	1 (ref)	1 (ref)

a) Separate multivariate models indicated for perceived health and disease indicators

b) Statistically significant COR values in bold

c) Variable not included in the model, because it was not statistically significant when assessed with other variables

d) The categories having COR values close to each other were combined in all analyses

In the multivariate ordinal logistic regression analysis, perceived health was no longer associated with pet ownership. When investigating which explanatory variables included in the model caused the disappearance of the statistical significance, basic education, form of housing, or BMI did so. The adding of gender, age, marital status, vocational education, or working in agriculture into the model one by one made no difference.

#### Sum of Disease Indicators

The association of pet ownership with the sum of disease indicators was about the same as in the case of perceived health with the cumulative odds ratio only slightly deviating from 1.0 (Table 5). Women had more diseases than men and working in agriculture was of no significance. BMI was the strongest health risk factor. Socio-demographic variables or individual health risk factors in the model did not weaken the association of having a pet with the sum of disease indicators. In the multivariate model that contained all background variables, a weak statistically significant association was observed between pet ownership and sum of disease indicators.

#### Dog or No Dog?

Among pet owners, 58% had dogs while 42% did not. Statistically significant associations of dog ownership were obtained with age, marital status, basic education, housing, and working in agriculture (p<0.001 each), and with vocational education at 0.2% risk level. People with dogs were most often those with couple relationships, having low basic or vocational education, living in single family homes, or working in agriculture.

In perceived health, dog owners did not differ from those not owning one. More dog owners than of those not having them had high cholesterol (p<0.001) or sciatica (p<0.001). They exercised strenuously, were frequently moving about, taking up sports, or in training, or were hunting, fishing, boating. Yet, 73% of the dog owners and 76% of those not having one had BMI less than 27 (p<0.001 for all).

In the ordinal multiple regression analysis, dog ownership was not associated with perceived health but was slightly associated with the sum of disease indicators (COR = 1.1; 95% CI: 1.0–1.2). The association faded away when individually controlling for gender, age, marital status, basic education, form of housing, hunting, fishing and boating, or BMI. An independent association was observed even after individually controlling for vocational education, cigarette smoking, use of alcohol, strenuousness of exercise, or by moving about, taking up sports, or being in training (COR = 1.1; 95% CI: 1.0–1.2 each).

## Discussion

BMI surfaced as the risk factor most strongly associated with pet ownership. Based on the multivariate analyses, findings involving positive associations of pet ownership with poor perceived health or disease indicators may be considered *directional* since the connections were weak. Together with low social class, a large BMI turned out to be a factor that brought about the fading away of the association of pet ownership with perceived health.

The database was large (N = 21,101) but the response rate was small (40%). The published analysis of non-response revealed no major selective health-related factors [24]. The non-respondent analysis used demographic and health-related population characteristics from the official statistics as well as behavioral, physical, and mental health-related outcome differences between early and late respondents to predict possible non-response bias. Non-respondents were likely to be men, older, single/divorced/widowed, and to have less education than the respondents had. The direction of error was such that the associations of the present study would have been even stronger providing the response rate had been greater. In a large pool, even small differences will easily produce statistically significant p-values. As compared with a national study, representativeness of the HeSSup Study was considered reasonable as far as good perceived health, heart disease, stroke, and diabetes were concerned [29].

Pets seem to be part of the lives of older people who have settled down and experience an increase in the number of illnesses, whereas young healthy single people have no time, need, or possibility for a pet. Associations of pet ownership with disease indicators were largely explained with socio-demographic factors; characteristics were those that bring forward the background of poor health in epidemiological investigations: male gender, low level of education, life without a couple relationship, and poor social standing [30].

One would-be mechanism between pet ownership and health has been that pet owners may have more vigorous exercise than others [1], [2], [7], [31]. The scientific relationship has not been clear, nonetheless [11], [16]. Substantial health gains and reductions in medical care expenses may be obtained providing all dog owners walk their own dogs and hence prevent diseases substantially [4]. The pet owners of the present study failed to exercise more than the rest. Dog owners moved about more than those having other pets but their greater BMI values let us to believe that they still have lost the margin on health that could have been produced with exercise by walking their own dogs.

Spontaneous exercise separate from work is more popular in the higher social classes [32]. Typical pet owners of these data are primarily regular people of humble means and set in their ways. Many of them work in agriculture and live on their single houses where having a pet is natural and possible because of the space to roam around. Poor social standing in life fails to utilize the exercise potential that contributes to sufficient health promoting resources or a disease reducing lifestyle. Exercise potential that exists because of pets may not be recognized as a means for health promotion in these groups [33], [34].

The desired associations of pet ownership with cardiovascular and other health risk factors were not demonstrated as other authors have indicated [6], [10]. High blood pressure has been noted among pet owners earlier [18] but comparisons with the previous studies are difficult. Some studies have been implemented with the help of volunteers [8], whereas others were based on follow-up designs [16] or used different methods of data collection [1]. Cross-sectional data do not allow examining the cause and effect relationships. Follow-up research with a carefully designed database will be needed for the real assessment as to what health and wellness role a pet plays, e.g., in the lives of sick and disabled or lonely people [1].

Except for the cholesterol issue, the associations of pet ownership and other cardiovascular health problems were not gender-related. Depression, panic attacks, migraine, and rheumatoid arthritis were more often associated with pet ownership among women. The associations of somatic diseases with pet ownership were more common among aging people, whereas psychiatric symptoms and diseases were more apparent among young people. The pressures of younger generations resulting from work or from combining work and family life may materialize as stress symptoms and be further emphasized in the relationships with the pets. Older generations begin to have more physical diseases but the pet ownership continues to be a shared hobby among family members. Physical abilities and functions of 50–54-year-olds are on the decline, and a pet may be perceived as being a difficult one to take care of then.

Although pets are expected to have propitious value in real life, the traditional health indicators will not coach it out. It may well be that a pet does not mend conditions diagnosed using medical criteria. Rather, a pet may help with the coping of difficult situations. It may well be that pets indeed contribute to positive health effects among people. We just do not know how the processes take place [12]. In the present study, a conceptual choice was made to examine depression and panic attacks as indicators of diseases. It is evident that depression will expose people to other diseases. It is thus possible to examine indicators of psychological states of illness or disease as risk factors of diseases.

Research has not advanced adequately to illustrate the medical conditions of influence or the processes of what might happen at the molecular level. The report of the *American Heart Attack Survey* [35]–[36] indicated that within a year following a coronary event, a less obvious risk of mortality was evident among pet owners than among those not having a pet. A potential mechanism may be that pet causes the brain to release endorphins that in turn will have a calming effect on the autonomic nervous system, and consequently, the lowering of the heart rate [37]. Taking care of a pet or talking to it may lower blood pressure and thus contribute to the beneficial effects [7].

Changes in the family structure and breaking down of the traditional communities may contribute to an upward trend in pet ownership [4], [38], [39]. Technological advances as a part of life may have raised the desire to have unselfish social relationships in return and to be close to nature and other living things [12], [40]. Hobbies involved with nature and wilderness may hint to this among pet owners. The emerging concepts of *trust* and *bonding* in social capital literature offer useful viewpoints for the examination of relationships between people and their pets [41], [42]. In addition to providing companionship, a pet may promote health by providing an option to play and relax and thus ease stress that can make people susceptible to illness or disease [43].

The fact that no health benefits of pet ownership were observed in the present cross-sectional population-based study may lead us to believe that the most important purpose or product of having a pet is not health-related, or does not indicate lack of disease as measured with the commonly known indicators. Other kinds of experiences and aspects of life may be involved and mental and emotional issues would need to be looked at. It may require focusing on special issues or population groups [44] as well as on using qualitative methodology in support of quantitative data.

### Conclusion

Pet ownership was very lightly associated with poor health in the general working-aged population when using several health and disease indicators. Pet owners had a slightly higher BMI than the rest, which indicates that people having a pet (particularly a dog) could use some exercise. A great challenge is awaiting public health workers in making a combined exercise and nutrition program for the kind of middle-aged population group that has established itself in life, has a low level of basic education, and owns the most pets, particularly living in rural locations. Investigation of effects generated by pet ownership is at the good but early beginning, and it is now important to establish studies with representative population based databases in order to test hypotheses involving effects of pet ownership and various health related dimensions within population groups that are composed of different kinds of background characteristics.
